# How supportive supervision influences immunization session site practices: a quasi-experimental study in Odisha, India

**DOI:** 10.3402/gha.v8.25772

**Published:** 2015-01-14

**Authors:** Bhuputra Panda, Sanghamitra Pati, Srinivas Nallala, Abhimanyu S. Chauhan, Anita Anasuya, Meena Som, Sanjay Zodpey

**Affiliations:** 1 Indian Institute of Public Health, Bhubaneswar, India; 2 Public Health Foundation of India, New Delhi, India; 3 DFID, Technical Management and Support Team, Government of Odisha, Odisha, India; 4 UNICEF State Office for Odisha, Bhubaneswar, Odisha, India

**Keywords:** immunization practices, supportive supervision, service delivery, session site, Odisha

## Abstract

**Background:**

Routine immunization (RI) is a key child survival intervention. Ensuring acceptable standards of RI service delivery is critical for optimal outcomes. Accumulated evidences suggest that ‘supportive supervision’ improves the quality of health care services in general. During 2009–2010, the Government of Odisha and UNICEF jointly piloted this strategy in four districts to improve RI program outcomes. The present study aims to assess the effect of this strategy on improvement of skills and practices at immunization session sites.

**Design:**

A quasi-experimental ‘post-test only’ study design was adopted to compare the opinion and practices of frontline health workers and their supervisors in four intervention districts (IDs) with two control districts (CDs). Altogether, we interviewed 111 supervisor–supervisee (health worker) pairs using semi-structured interview schedules and case vignettes. We also directly observed health workers’ practices during immunization sessions at 111 sites. Data were analyzed with SPSS version 16.0.

**Results:**

The mean knowledge score of supervisors in CDs was significantly higher than in intervention groups. Variegated responses were obtained on case vignettes. The control group performed better in solving certain hypothetically asked problems, whereas the intervention group scored better in others. Health workers in IDs gave a lower rating to their respective supervisors’ knowledge, skill, and frequency of supervision. Logistics and vaccine availability were better in CDs.

**Conclusion:**

Notwithstanding other limitations, supportive supervision may not have independent effects on improving the quality of immunization services. Addressing systemic issues, such as the availability of essential logistics, supply chain management, timely indenting, and financial resources, could complement the supportive supervision strategy in improving immunization service delivery.

Supportive supervision (SS) as a strategy ensures that personnel carry out their activities effectively through direct, personal contact on a regular basis; this would guide and support peripheral functionaries to develop professional competence (1). Correctly followed, SS is an effective mechanism for enhancing the quality of services (2). SS strengthens relationships within the system, focuses on the identification and resolution of problems on the site, optimizes resource allocation, and promotes teamwork and two-way communication (3).

Different supervision strategies influence performance. Effective and regular supervision could potentially help meet the challenges unique to health workers, especially in the context of task-shifting initiatives that transfer tasks from health supervisors to health workers (4–6) . Other studies indicate that adequate supervision is considered key to ensure that health workers perform well, are motivated, and have well-defined roles in the community and in relation to the health system (7–10) . Exploratory studies have consistently identified quality supervision as a positive contributor to community health workers’ job motivation, retention, and satisfaction (11). If done poorly, supervision can also contribute to dissatisfaction (12). Supervision by health supervisors gives health workers a sense of legitimacy in the eyes of other staff, the communities they serve, and themselves (13, 14).

SS usually involves record reviews, observations, performance monitoring, constructive feedback, provider participation, and problem solving. In practice, SS strategies vary greatly in approach, content, and tools (15). Despite the recognized role that SS can play in performance and motivation, numerous studies from a range of countries and programs have found that supervision often has low coverage and low administrative focus; is irregular, unsupportive, and demotivating; and lacks adequate training for supervisors and problem solving or feedback mechanisms for providers (15–19) .

The national universal immunization program (UIP) review (2004) and vaccine management assessment tool (VMAT) study (2007) identified inadequate SS and insufficient training (in terms of the quality and number of health workers trained) by the government as foremost gaps underlying poor coverage and quality of immunization (20, 21). Consequently, the Government of Odisha in partnership with UNICEF initiated a pilot intervention, ‘Routine Immunization Assessment cum Training Workshops on Supportive Supervision’, for the supervisors of a routine immunization (RI) program in four priority districts, namely Bolangir, Koraput, Malkangiri, and Nawarangpur. This intervention was implemented during August 2009–February 2010. About 8–10 participants from each block of these districts attended this training in batches. The overall topics consisted of subjects such as development of SS guidelines for district immunization managers, district-level training in continuous SS, monitoring and evaluation of performance, and allocation of resources for district managers to cover travel and communication costs. SS, which was the focal point of the package of interventions, was based on 1) introducing updated job descriptions with documented lines of supervision; 2) improving communication lines and skills; 3) introducing guidelines and tools for supervision, performance review, and monitoring; and 4) evidence-based action planning.

Full-scale implementation of this pilot initiative was reported since May 2010. The subsequent 12 months involved extensive monitoring and on-the-job training of immunization managers and supervisors to improve supervision practices and to help providers solve immunization-related problems. Immunization managers from four intervention districts (IDs) were encouraged to apply SS guidelines in practice. Guidelines and tools for supervision included instructions for conducting supervision, namely rules of conducting supervision meetings, checklists for supervisory visits, work planning action sheets, do's and don'ts of supervision, self-assessments of supervisors’ competencies, tips on delegation, a feedback mechanism, and conflict resolution. It was mandatory for every immunization manager from the intervention group to visit sessions at least once a week during the fixed outreach session. The category of participants were medical officers (MO), AYUSH medical officers (AYUSH MO), block extension educators (BEE), block program organizers (BPO), lady health visitors (LHV), integrated child development scheme(ICDS) supervisors, and supervisors at the block level of the women and child development (WCD) department.

The overall aim of this study was to assess the effect of a ‘supportive supervision’ strategy on the quality of immunization services. It is a comparative study between four IDs and two non-intervention (control) districts. The specific objectives were 1) to assess and compare the attitudes and practices of supervisee–supervisor pairs toward each other, acceptance of the SS strategy, problem-solving skills, management capacity, the communication process, and on-site correction abilities and 2) to recommend to the state government and to UNICEF the usefulness of the SS strategy for improvement of quality of services.

## Design

### Study design and settings

A quasi-experimental ‘post-test only’ study design was used (22, 23). The data collection captured information on technical knowledge, role clarity, and practices at the immunization site of supervisors and supervisees. The roles and responsibilities of supervisors and supervisees are summarized in Table 1.

**Table 1:** Role of supervisees and supervisors in RI session

Type of role	Role of supervisee	Role of supervisor
Organizing sessions	Finalizing the beneficiaries’ list Mobilizing beneficiaries Indenting vaccines, drugs and other logistics Organizing the fixed monthly RI outreach sessions	Ensure sessions are conducted as per the plan Make supervisory visits to sessions
Service delivery	Primary Immunization Boosters Tetanus toxoid for Pregnant women Vitamin A supplementation IFA supplementation Deworming	Monitor Hand-holding support Problem solving Fact-finding and validation
Health education	Imparting education on a specific health topic through group counseling Addressing individual health issues through individual counseling	Make supervisory visits Ensure that complete and accurate messages are given Provide need-based guidance
Reporting	Preparing session-wise reports Submitting reports to the supervisor	Collect and analyze reports Provide feedback

Four pre-existing IDs and two comparable control districts (CDs) were taken for data collection. Selection of CDs was performed on the basis of comparable baseline UIP indicators as of December 31, 2009, through desk review: we considered complete immunization coverage, adverse events following immunization (AEFI) cases reported, and dropout rates for such comparison. On the basis of geographic, sociopolitical, and programmatic similarities, Kandhamal and Kalahandi were taken as CDs for data collection.

### Operational definitions

1. Link workers, also known as auxiliary nurse and midwives (ANMs), constituted the supervisees in this study. They are also termed as ‘end-users’ or ‘multipurpose health worker – female’ (MPHWF). Supervisory staff who had undergone the SS training constituted the universe of supervisors. All supervisors who had undergone the SS training but had been transferred or retired during the study were excluded from the study.
2. Quality is synonymous with innate excellence (e.g. attainment of superiority, becoming useful) (24, 25). There is no universally accepted definition of quality. Lohr et al. define quality as ‘the degree to which health services for individuals and populations increase the likelihood of desired health outcomes and are consistent with current professional knowledge’ (26). We are of the view that good quality of services means provision of client-centric, appropriate services in a cultural acceptable manner by technically competent service providers who have good communication and decision-making skills (27, 28).

SS is defined as a process that promotes quality at all levels of the health system by strengthening relationships within the system, focusing on the identification and resolution of problems, and helping to optimize the allocation of resources promoting high standards, teamwork, and better two-way communication (29). We consider SS comprising any support provided by the internal supervisors and external monitors toward improvement of service delivery and effective implementation of the immunization program.

### Sampling

The primary outcome considered for calculating sample size was a composite of practices (score>2) of supervisors with regard to immunization session site supervision. With 95% confidence level and 80% power, expected frequency of 70% and worst acceptable of 50%, we calculated 16 RI sessions in each district to be sufficient for data collection. A buffer of 20% was added to the total sample to compensate for possible non-responses. Therefore, a minimum of 102 sessions and a maximum of 122 sessions were planned for directly observing sessions and interviewing supervisees as well as supervisors. We observed 111 immunization sessions (ID=72; CD=39) and interviewed an equal number of supervisors and supervisees. The session sites were randomly selected using a lottery method. It is important to mention here that in Odisha, RI sessions are conducted as outreach services only once a week on Wednesday. To ensure representation of each category of supervisors, a quota sampling method was adopted. In consultation with the district administration, our field investigators accompanied the selected sample supervisors at least once to the session site during data collection.

### Data collection

Data were collected from April to December, 2011. Semi-structured interview schedules of supervisors and supervisees (each) were first developed in English, translated into the local language, and field-tested in three non-sample sessions and with six supervisor–supervisee pairs. The supervisors’ interview schedule contained both structured multiple-choice responses and open-ended hypothetical situations, whereas the supervisees’ interview schedule focused on the frequency of supervisory visits and the body language, communication style, knowledge level, attitude, and problem-solving skills of supervisors. We used a direct observation checklist recommended by the Government of India for on-site observation of session sites. It had questions related to the micro-plan, the availability of logistics and supplies, and practices directly related to the quality of immunization, such as needle touching, waste management, vaccine returning practices, use of cards, delivery of key messages, and so on. The researchers and supervisors (internal monitors) separately collected the data from the same session, using the same tool. To avoid bias in data collection, the supervisor was not informed beforehand about the need to collect such information. We found all supervisors carried the supervisory checklist when visited the session along with the researcher; after the session visit, the researcher collected a photocopy of the checklist from the supervisor. To ensure uniformity, six field investigators were hired and trained on the data collection tools for data collection. A team led by the principal researcher monitored the quality of data collection through regular field visits and cross-checking.

### Data analysis

Quantitative data were entered into Microsoft Excel and then exported to SPSS version 16.0 for analysis. Questions on attitude scales were analyzed through derivation of means; statistical techniques, such as chi-square and *t*-tests, were used to infer the significance of associations and differences.

### Ethical issues and quality assurance

We obtained ethical approval from the institutional ethical committee of the Indian Institute of Public Health – Bhubaneswar (IIPHB). Confidentiality and anonymity were maintained throughout the data collection process. Informed consent of all respondents was taken before instituting the interview schedules. Written consent of all respondents was obtained, and they were briefed about the study objectives. The respondents were free not to respond to any question and were free to leave the study at any stage.

### Limitations

The main limitation of the study is non-availability of baseline data, which makes it difficult to attribute the results to the intervention. There is the possibility that a modest role of intervention in improving service outcomes could be due to a type II error, resulting from an inadequate number of districts (we chose two CDs against four IDs). Finally, the short duration of the intervention may have restricted its potential to bring the expected outcomes.

## Results

Supervisors’ interviews revealed that there are significant differences in their understanding of the basic principles behind SS. The interview schedule of supervisors had both positive and negative questions. On questions related to the importance of supervisors motivating supervisees, the role of punitive measures in SS, and the importance of supervision in the improvement of supervisees’ performance, the mean score of CDs was significantly higher than that of intervention groups (Table 2). On other knowledge-related questions, such as the need to seek information from supervisees, the role of supervisors in the planning process, the role of supervisors in providing updated information to supervisees, and the process of pointing out the mistakes of supervisees during immunization sessions, CDs had better mean scores than IDs, although it was statistically not significant. However, with respect to the actual practice of supervisors on key supervision issues, it was found that IDs had better mean scores than CDs (Table 3).

**Table 2:** Knowledge and attitude of supervisors in IDs and CDs

Attributes	ID N=72	CD N=39	Sig.
Motivating supervisee is an important function of RI supervision	4.53	4.90	0.000
Punishment is sometimes required during supportive supervision	1.99	1.85	0.05
Seeking information from the supervisee is important for problem solving	4.33	4.51	0.369
Supervision is important to improve supervisee's performance	4.43	4.85	0.000
Supervision provides information for planning at all levels	4.25	4.51	0.715
Relevant new information on RI should not be given directly to the health worker during supervision	2.69	3.08	0.234
All mistakes should be immediately pointed out to the ANM in the presence of community members during the session	2.07	1.62	0.869

ID: intervention district; CD: control district; RI=routine immunization; ANM=auxiliary nurse and midwife.

For all the items mentioned in the table, the Likert scale ranged from 1 to 5, where 1=strongly disagree and 5=strongly agree.

**Table 3:** Practice of supervisors in intervention and control districts

Attributes	ID *N*=72	CD *N*=39	Sig.
Do you give prior information to health workers before you visit supervision?	2.38	1.85	0.059
Do you undertake revisits to supervise the same health worker?	2.13	2.08	0.131
Do you find out and visit the priority blocks?	2.22	2.36	0.466

For all the items mentioned in the table, the Likert scale ranged from 1 to 3, where 1=rarely, 2=sometimes, and 3=usually.

**Table 4:** Supervisors’ problem-solving skills on hypothetical situations

Attributes	Type of district	*N* a	Mean desirable response	Std. deviation	Std. error mean
What if fully melted icepacks are found?	ID	72	1.1	0.858	0.101
	CD	39	1.54	0.884	0.142
Is waster disposed at session site?	ID	69	1.17	0.804	0.097
	CD	39	0.95	0.759	0.122
Is ANM not returning unused vials?	ID	72	0.75	0.687	0.081
	CD	39	0.87	0.57	0.091
Is ANM injecting DPT in the gluteal region?	ID	72	1.79	1.404	0.165
	CD	39	0.77	0.986	0.158
Has the ANM not mentioned reconstitution time?	ID	72	1.26	1.061	0.125
	ID	39	0.95	0.857	0.137
Is ANM not giving four key messages?	ID	72	1.99	1.204	0.142
	CD	39	2.31	1.195	0.191
Is ANM touching needles?	ID	72	1.39	0.881	0.104
	CD	39	1.05	0.759	0.122
Are skills among functionaries adequate?	ID	72	1.5	1.035	0.122
	CD	39	0.92	0.739	0.118
Is ANM putting cut syringes in red bag?	ID	71	1.11	1.45	0.172
	CD	39	2.62	1.016	0.163
Is correct dose of Hepatitis B given?	ID	70	1.63	1.505	0.18
	CD	25	1.04	1.369	0.274
Is ANM attending session late?	ID	71	0.62	0.763	0.091
	CD	32	0.91	1.118	0.198
Is ANM photocopying the passbook?	ID	63	1.03	1.015	0.128
	CD	38	1.53	1.202	0.195

ID=intervention district; CD=control district.

a *N* has been computed after excluding non-responses.

When asked about hypothetical situations to assess the problem-solving abilities of supervisors in a series of open-ended questions, we received variegated responses. A joint meeting of UNICEF representatives, IIPHB faculty, and senior officials of the directorate of family welfare, Government of Odisha, decided to rank the responses on a scale of 0–3, wherein 0 represents a totally wrong answer, 1 is a partially correct but unacceptable answer, 2 is an incomplete but correct answer, and 3 means a fully correct answer. Analysis of the findings indicated that on issues related to waste disposal, correction of site of a DPT (diphtheria, pertussis, and tetanus) injection, reconstitution of diluents, correction of needle touching, improvement of skills among health staff, and rectification of doses of hepatitis B vaccine, the supervisors of IDs had significantly higher levels of correct responses as compared to those of CDs.

In contrast, when asked about the ways of dealing with melted icepacks, return of unused vials, key messages at immunization sites, use of red bags for waste disposal, late arrivals of ANMs for sessions, and photocopying the passbook, CDs had better responses (Table 4). Levene's test for equality of variance (Table 5) revealed that supervisors of IDs had significant correct responses with respect to two questions, whereas CD supervisors had significant correct responses toward another two questions.

**Table 5:** Independent samples test on supervisors’ knowledge and practices^a^

	Levene's test for equality of variances		*t*-test for equality of means		
	
Attributes	*F*	Sig.	*t*	df	Sig. (2-tailed)
What if fully melted icepacks are found?	1.349	0.248	−2.559	109	0.012
Is waster disposed at session site?	0.654	0.42	1.427	106	0.157
Is ANM not returning unused vials?	4.218	0.042	−0.944	109	0.347
Is ANM injecting DPT in the gluteal region?	39.919	<0.001	4.038	109	<0.001
Has the ANM not mentioned reconstitution time?	6.205	0.014	1.593	109	0.114
Is ANM not giving four key messages?	0.985	0.323	−1.346	109	0.181
Is ANM touching needles?	9.823	0.002	2.02	109	0.046
Are skills among functionaries adequate?	20.21	<0.001	3.08	109	0.003
Is ANM putting cut syringes in red bag?	41.555	<0.001	−5.74	108	<0.001
Is correct dose of Hepatitis B given?	13.895	<0.001	1.717	93	0.089
Is ANM attending session late?	3.849	0.053	−1.517	101	0.132
Is ANM photocopying the passbook?	8.7	0.004	−2.211	99	0.029

a Equal variance assumed.

**Table 6:** Ranking by supervisees on program skills of supervisors in IDs and CDs

		Intervention category		
			
Process indicators of supportive supervision		ID (*N*=72) number (%)	CD (*N=*39) number (%)	Total (*N=*111) number (%)
Number of supervisory visits in the past 6 months	Once	15 (22.2)	18 (46.2)	34 (30.6)
	Two to three times	28 (37.5)	7 (17.9)	34 (30.6)
	More than three times	29 (40.5)	14 (35.9)	43 (38.7)
Type of communication	One way	22 (30.6)	9 (23.1)	31 (27.9)
	Two ways	50 (69.4)	30 (76.9)	80 (72.1)
Checklist availability	Yes	50 (69.4)	32 (82.1)	82 (73.9)
	No	22 (30.6)	7 (17.9)	29 (26.1)

ID: intervention district; CD: control district.

Through interviewing the supervisees, we assessed the number of supervisory visits made in the past 6 months, and the communication styles and on-site correction practices of supervisors. We found that 40.5% of supervisors in IDs and 35.9% of supervisors in CDs had more than three visits to the session in the past 6 months. Two-way communication was higher in CDs (76.9%) as compared to the IDs (69.4%), although this was not statistically significant. With respect to the availability of session-monitoring checklists, we found the CDs had better results than IDs. Furthermore, as many as 15 out of 72 and 18 out of 39 sample supervisees reported that their supervisors had visited the session site only once during the past 6 months in intervention and CDs, respectively (Table 6).

**Table 7:** Ranking by supervisees on knowledge, attitude, and problem-solving skills of supervisors

		Intervention category			Mean		
				
Process indicators of supportive supervision		ID (*N*=57)^a^ number (%)	CD (*N*=21)^b^ number (%)	Total (*N*=111) number (%)	ID	CD	Sig.
Attitude of supervisor	Not applicable	15 (20.8)	18 (46.2)	33 (29.7)	1.93	2.14	0.16
	Good	9 (12.5)	5 (12.8)	14 (12.6)			
	Very good	43 (59.7)	8 (20.5)	51 (45.9)			
	Excellent	5 (6.9)	8 (20.5)	13 (11.7)			
Knowledge of supervisor	Not applicable	15 (20.8)	18 (46.2)	33 (29.7)	2.74	3	0.144
	Poor	1 (1.4)	0	1 (0.9)			
	Good	19 (26.4)	6 (15.4)	25 (22.5)			
	Very good	31 (43.1)	9 (23.1)	40 (36.0)			
	Excellent	6 (8.3)	6 (15.4)	12 (10.8)			
Problem-solving skills	Not applicable	15 (20.8)	18 (46.2)	33 (29.7)	2.53	3	0.009
	Poor	1 (1.4)	0	1 (0.9)			
	Good	28 (38.9)	7 (11.9)	35 (31.5)			
	Very good	25 (34.7)	7 (17.9)	32 (28.8)			
	Excellent	3 (4.2)	7 (17.9)	10 (9.0)			

ID: intervention district; CD: control district.

a 15 not applicable in IDs.

b 18 not applicable in CDs.

In the second category of questions, assessments of the knowledge, attitude, and problem-solving skills of supervisors were ranked by the supervisees on a scale of 0–4 (0=not applicable; 1=poor; 2=good; 3=very good; 4=excellent). This question was asked only to those supervisees whose supervisors visited the session sites more than once in the past 6 months. An analysis of results revealed that supervisees of CDs ranked their supervisors better than by the IDs, although this was statistically not significant (Table 7). One possible explanation to this could be that in IDs, the expectation of supervisees from their respective supervisors was higher than in CDs.

**Table 8:** Ranking by supervisees on the management skills of supervisors in IDs and CDs

		Intervention category			Mean		
				
Process indicators of supportive supervision		ID (*N*=57)^a^ number (%)	CD (*N*=21)^b^ number (%)	Total (N=111) number (%)	ID	CD	Sig.
Body language of supervisor	Responsive	44 (61.1)	27 (69.2)	71 (64.0)	2.6	2.64	0.68
	Reflective	27 (37.5)	10 (25.6)	37 (33.3)			
	Fugitive	1 (1.4)	2 (5.1)	3 (2.7)			
Communication process	Authoritative	1 (1.4)	3 (7.7)	4 (3.6)	3.65	3.36	0.04
	Supportive	52 (72.2)	21 (53.8)	73 (65.8)			
	Friendly but not supportive	17 (23.6)	13 (33.3)	30 (27.0)			
	Not involved	2 (2.8)	2 (5.1)	4 (3.6)			
Process of correction	Fault finding	15 (20.8)	3 (7.7)	18 (16.2)	2.51	2.49	0.918
	Non-explanatory	8 (11.1)	5 (12.8)	13 (11.7)			
	Supportive	26 (36.1)	14 (35.9)	40 (36.0)			
	Does not react	23 (31.9)	15 (38.5)	38 (34.2)			
	Any other	0	2 (5.1)	2 (1.8)			

ID: intervention district; CD: control district.

a Not applicable=15 in IDs.

b Not applicable=18 in CDs.

The researchers ranked the body language, communication process, and correction process of the supervisors at the session site on the basis of the response given by the supervisees. Table 8 indicates that the IDs had better scores than the CDs with regard to the first two attributes. However, with respect to the process of correction, both groups had very similar scores. This result may be cautiously inferred, since 18 respondents were missing from the sample of CDs.

Through direct observation of immunization session sites, we assessed the regularity in holding sessions, availability of logistics, and practices of supervisees. Availability of antigens and other logistics in CDs was better than in IDs, although such differences were statistically not significant. One of the probable explanations behind such unexpected results is that decentralization as a policy directive has not yet percolated down to the district and sub-district levels. Furthermore, the availability of supplies and logistics is a function of sufficient availability of stock at central and block stores, which is dependent upon timely indenting and receipt of items. Consequently, we infer that SS training had not improved the skills of supervisors with respect to these management functions. We wanted to cross-verify these findings with data triangulation. Thus, direct observation results of the supervisor was analyzed, and it was found that similar, rather significant differences existed in the reports of supervisors with regard to the availability of supplies and logistics in immunization sessions. Significant differences were found with regard to their reports on the practice of alternate vaccine delivery (AVD); the availability of measles vaccines, oral rehydration solutions (ORSs), blood pressure apparatus, black and red bags, DPT vaccines, and iron folic acid (IFA) tablets; and their compliance with the micro-plan. In the next step, we analyzed the difference in observation of findings on key practices in IDs and CDs. Results indicated a mixed response. For instance, with regard to the use of hub cutter, delivery of four key message, such as next immunisation date, possible side effects, how to address the side effects and the importance of mother and child protection card, and proper handling of syringes by the ANMs, IDs performed better than CDs. However, such differences were statistically not significant.

We analyzed the differences in the findings of the researcher and the supervisor to better understand the accuracy of reporting by the supervisors, who are also called ‘internal monitors’. To ensure neutrality in reporting, the researchers were specifically trained to objectively record the findings from the session. Results revealed that there were notable differences with respect to reporting of each and every item, although both researcher and supervisor were using the same checklist. Therefore, objectivity in session assessment by the supervisors was not maintained, and individual differences in perspectives of reporting existed. The difference was significant with respect to reporting on the availability of black and red bags, the weighing machine, BP apparatus, the use of hub cutter, and four key message delivery. All of these items were over-reported by the supervisors as compared to their actual status in the sessions.

## Discussion

The results of the study suggest that the intervention package, which included SS guidelines, district-level trainings, continuous supervision and support during a 12-month period, monitoring of provider performance, and resource allocation for travel and communication of supervisors. The intervention independently contributed to improved knowledge of SS among supervisees, and it helped in removing self-perceived barriers such as availability of resources, a lack of formats for field visits, and a lack of recognition among providers regarding the importance of SS. Discussion with district officials indicated that they attached high importance to the utility of supervision as a key strategy to improve immunization quality. Given that most of the important public health program implementation is being performed by health workers, stationed in peripheral health posts and health centers, faulty supervision by higher officials could pose daunting management challenges at the block and district levels. One possible explanation of the results shown in Table 2 could be that the IDs had abysmally low levels of baseline understanding and that the intervention had improved their understanding but not up to the level of CDs. However, this assumption could not be cross-verified due to the absence of any baseline measurement of these attributes.

Over the past 10 years, we have noticed the establishment of new peripheral facilities; usually, the facilities are remote, have poor communication with the rest of the health care system, lack even basic supplies and equipment, and are staffed by under-trained and poorly paid personnel who typically work by themselves. Motivation is hard to maintain in such an atmosphere. Regular supervision of sessions, ILR points, and vaccine stores is an important step to ensuring quality immunization services. While supervision can be a very participatory process, traditional supervisory visits focus more on inspection and fault finding rather than on problem solving to improve performance. If health workers don't receive sufficient guidance or mentoring on how to improve their performance, they will remain undirected.

Other similar studies have pointed out the necessity of improving supervisory support to these facilities 30–32) . However, only a few intervention studies have been carried out to see how this could be accomplished practically, while addressing the cost of supervision. In developed countries, a recent review of randomized trials at selected facilities concluded that quality of care could be improved by continuing medical education, particularly if it included chart audits and other follow-up procedures (32). With health system decentralization, district supervisors are increasingly the only human contact between health workers in remote villages and the rest of the formal health care system (33).

Traditional approaches to performing supervisory visits are effective to an extent, but with several limitations: the supervisors tend toward facility inspection rather than human capital development. Often, supervisors themselves lack skills, tools, and resources and are over-burdened with other administrative duties on a daily basis. This needs to be addressed at the district level with utmost sincerity. There is very limited evidence on the subject in India. However, the benefits of supervision, as reflected in some of the published literature, include: helping service providers to achieve work objectives by improving their performance, ensuring uniformity to set standards, identifying problems and solving them in a timely manner, making a follow-up on decisions reached during a previous supervision visit, identifying staff needs and providing opportunities for personal development, and reinforcing administrative and technical links between higher and lower levels (34, 35).

There are institutions that provide training on performance improvement, but governments have not seriously dwelled on this option mainly for two reasons: first, investing in long-term capacity-building measures pre-necessitates the deputation of peripheral health staff for a longer period of time; and, second, although there are many examples and case studies where SS has been used to improve health worker performance and immunization coverage, long-term and sustainable results have not been thoroughly documented.

The large-scale health care reforms of the past decade included the need to ensure that immunization managers carry out their activities effectively. Through regular and personal contact, supervisors were mandated to improve the competence of peripheral health workers. However, recent reports are non-conclusive regarding the effects of such reforms (36). With the addition of the National Rural Health Mission (NRHM), renamed as the National Health Mission (NHM), service supply responsibilities and decentralization of health care financing were ensured, but delegation of decision-making powers and lines of responsibilities remained unclear. Consequently, supervisors at the sub-district level were helpless to address logistics and supply chain management issues. Although at the national level the program got expanded, coverage rates remained poor over the course of the reforms.

In Odisha, peripheral health staff are responsible for providing primary health care services, where supervision continues to be a problematic issue. Lack of experienced and quality human resources can easily jeopardize the success of any health program, including immunization (37). RI is a resource-intensive program, and there are many determinants to ensure its successful implementation, such as adequacy in financing, vaccine quality, vaccination practices, and strength of the health system. Therefore, strengthening supervision with a ‘mentoring’ spirit is the need of the hour (38).

Reporting by supervisors was not objective, and there were significant variations with regard to reporting on the availability of logistics and instances of undesirable activities. However, whether or not such distortions in reporting by supervisors were deliberate needs further scrutiny. There was a general improvement in the coverage of immunization services in both groups of districts. This can be attributed to improved knowledge of supervisees and increased acceptance of on-site corrections made by supervisors. The trend in improvement of service outcomes in both IDs and CDs can be attributed to other factors, such as an overall improvement in health care financing after the advent of NHMs and targeted service provision to the ‘hard-to-reach’ areas. Allocating resources to supervision is likely to result in improved performance of health workers.

SS can have independent positive effects on improvement of the knowledge and practices of supervisors and supervisees related to conducting immunization sessions. However, whether or not it has any conclusive effect on improvement of the overall program outcomes, because of the existence of many intermediate factors in the chain of events, needs further investigation. Thus, SS as a strategy may be conditionally envisaged for quality improvement within the overall framework of a national immunization program. Better health system preparedness is a necessity for the success of this strategy (39). As reflected in Table 6, we highlight the need to reinforce the mechanism of SS through repeated orientation of district- and state-level officials. One of the key systemic bottlenecks is a shortage of supervisory staff to cover so many sessions on a regular basis; this could be addressed partly through delegation of responsibilities at the block level and strengthening the micro-plan of supervisory visits.

## Conclusion

The old paradigm that most performance problems can be solved by training alone needs to be reconsidered. Further delineation of roles, mandatory provision for supervisory visits, the allocation of resources for field trips, and monthly reviews at the district and sub-district levels are key district-level interventions for SS to work. SS would require staff time and travel and per diem costs for supervisors. In the Odisha context, the health budgets frequently either do not allocate sufficient funds to conduct SS or do not transfer funds to peripheral facilities; this makes the job of supervisors difficult to finance and coordinate his or her visit: this may be addressed at district review meetings. Furthermore, supervisors need support and authority from the district to implement supervision or recommend context-specific changes to improve services in the periphery. Since the ranking score of supervisors with regard to communication skills, problem-solving skills, and frequency of supervisory visits was suboptimal, it is recommended to have modular training programs. An RI program also requires the active involvement of accredited social health activists (ASHAs) and anganwadi workers (AWWs). Therefore, establishing a platform of intersectoral convergence at the block and district levels could be crucial to discuss and address the systematic bottlenecks.

## Authors' contributions

BP conceptualized the study design, wrote the manuscripts, supervised the data collection, and conducted data analyses. SP contributed to the finalization of study tools, data analysis, and manuscript writing. SN contributed to development of study tools, data analysis, and manuscript writing. ASC undertook the literature review and writing of the data analysis. AA finalized the study design, data collection tools, and manuscript editing. MS helped in data collection, analysis, and writing. SZ provided technical support to finalize study design, data analysis, and manuscript editing.
